# Risk Analysis in the Lower Silesia Healthy Donors Cohort: Statistical Insights and Machine Learning Classification

**DOI:** 10.3390/jcm14248624

**Published:** 2025-12-05

**Authors:** Przemysław Wieczorek, Magdalena Krupińska, Patrycja Gazinska, Agnieszka Matera-Witkiewicz

**Affiliations:** 1 Screening of Biological Activity Assays and Collection of Biological Material Laboratory, Wroclaw Medical University Biobank, 50-556 Wroclaw, Poland; magdalena.krupinska@umw.edu.pl; 2 Biobank Research Group, Lukasiewicz Research Network—PORT Polish Center for Technology Development, 54-066 Wroclaw, Poland

**Keywords:** metabolic syndrome, risk stratification, machine learning, CatBoost, SHAP, systolic blood pressure, triglycerides, HDL cholesterol, population cohort, Poland

## Abstract

**Background/Objectives**: Metabolic syndrome (MetS) increases the risk of type 2 diabetes and cardiovascular disease. We aimed to identify the key metabolic predictors of MetS in a Central European cohort and to compare classical statistics with modern machine learning (ML) models. **Methods**: We analysed 956 adults from the Lower Silesia Healthy Donors cohort. Clinical, anthropometric, biochemical, and lifestyle variables were collected using standardised procedures. Group differences were tested with Mann–Whitney U tests and effect sizes. A multivariable logistic regression (outcome: binary MetS defined as ≥3 harmonised components, MetS_bin) estimated adjusted odds ratios. In parallel, ML models (logistic regression, Random Forest, XGBoost, LightGBM, CatBoost) were trained with stratified 5-fold cross-validation. Performance was evaluated by accuracy, F1-macro, and area under the receiver-operating characteristic curve (ROC AUC). Model interpretability used SHAP values. **Results**: Overweight/obese participants had higher fasting glucose (median 92.0 vs. 84.6 mg/dL), fasting insulin (9.9 vs. 6.6 µU/mL), and systolic blood pressure (134 vs. 121 mmHg) and lower HDL cholesterol (53 vs. 66 mg/dL) compared to normal-BMI individuals (all *p* < 0.001, r ≈ 0.39–0.41). Participants with a higher waist circumference also showed markedly increased HOMA-IR (2.16 vs. 1.34; *p* < 0.001). In multivariable logistic regression, waist circumference, BMI, triglycerides, HDL cholesterol, fasting glucose, and systolic blood pressure were independently associated with MetS, yielding a test ROC-AUC of 0.98 and PR-AUC of 0.88. Machine learning models further improved discrimination: Random Forest, XGBoost, LightGBM, and CatBoost all achieved very high performance (test ROC-AUC ≥ 0.99, PR-AUC ≥ 0.98), with CatBoost showing the best cross-validated PR-AUC (~0.99) and favourable calibration. SHAP analyses consistently highlighted fasting glucose, triglycerides, HDL cholesterol, waist circumference, and systolic blood pressure as the most influential predictors. **Conclusions**: Combining classical regression with modern gradient-boosting models substantially improves the identification of individuals at risk of MetS. CatBoost, XGBoost, and LightGBM delivered near-perfect discrimination in this Central European cohort while remaining explainable with SHAP. This framework supports clinically meaningful risk stratification—including a “subclinical” probability zone—and may inform targeted prevention strategies rather than purely reactive treatment.

## 1. Introduction

Metabolic syndrome (MetS) is a cluster of interrelated metabolic abnormalities that substantially increase risk of cardiovascular diseases and type 2 diabetes [1,2]. According to the International Diabetes Federation (IDF) and the American Heart Association/National Heart, Lung, and Blood Institute (AHA/NHLBI), MetS is diagnosed when at least three cardiometabolic risk factors are present, typically involving central obesity, dyslipidemia, elevated blood pressure, and impaired glucose regulation [3,4]. The prevalence of MetS has grown significantly in recent years, representing a critical public health challenge worldwide [1,2]. The International Diabetes Federation (IDF) and the American Heart Association/National Heart, Lung, and Blood Institute (AHA/NHLBI) provide widely recognised definitions of MetS. In addition, the European Society of Hypertension (ESH) and European Society of Cardiology (ESC) highlight the importance of assessing overall cardiovascular risk in individuals with MetS, recommending tools such as SCORE2 for personalised risk stratification [5].

The growing prevalence of MetS shows substantial regional variability. Central and Eastern Europe exhibit some of the highest documented rates [6,7,8]. In Poland, the WOBASZ II study (2013–2014) estimated that MetS affects approximately 33% of women and 39% of men, demonstrating an upward trend over time [6,7]. More recent multicenter research among Polish women aged ≥ 35 years without prior cardiovascular disease, diabetes, or chronic kidney disease confirmed that over 20% met the diagnostic criteria for MetS, with prevalence significantly higher among those with obesity; physical activity was associated with a lower risk of developing MetS and its components [6,8]. These findings align with broader epidemiological trends in Europe, where MetS prevalence varies considerably. In Western Europe, studies report rates ranging from approximately 15% in France to 34% in Finland and Italy [2,8]. Such regional differences are typically attributed to variations in lifestyle, diet, healthcare systems, and genetic predisposition. Notably, Southern and Northern European populations present some of the highest rates, reflecting the complex interplay of environmental factors and metabolic risk.

The burden of MetS extends beyond prevalence, as it significantly elevates the risk of adverse health outcomes, including type 2 diabetes, cardiovascular events, and all-cause mortality. Studies indicate that MetS amplifies the risk of myocardial infarction by 2.5-old, increases overall mortality by 1.5-fold, and nearly doubles the likelihood of cardiovascular incidents [1,2]. Given these risks, population-specific assessment of metabolic factors—particularly in Central and Eastern Europe—remains essential for developing targeted prevention and intervention strategies.

Lower Silesia, a socio-demographically diverse region of Poland, offers a unique setting for investigating metabolic risk. The Lower Silesia Healthy Donors cohort provides comprehensive health data, enabling detailed statistical analyses and the identification of potential relationships between metabolic parameters and MetS risk. The integration of regional data with global trends allows for a nuanced understanding of MetS, highlighting the relevance of characterising metabolic patterns in Central European populations, which remain underrepresented in international research.

This study aims to provide a clinically grounded evaluation of metabolic syndrome (MetS) by identifying the metabolic determinants most strongly associated with the presence of MetS (MSS ≥ 3), reflecting the real diagnostic definition used by the IDF and AHA/NHLBI. Unlike prior studies using continuous severity scores or non-clinical composite indices, this work focuses on predicting MetS as a binary, clinically actionable outcome.

Furthermore, this study systematically compares classical statistical modelling with state-of-the-art machine learning approaches to evaluate their relative performance in identifying individuals at risk [9,10,11,12,13,14]. By integrating detailed epidemiological data from the Lower Silesia Healthy Donors cohort with current international evidence, this research introduces a novel, population-based perspective from Central Europe and evaluates the interpretability of ML models using SHAP values in a real clinical context [15,16]. Ultimately, the findings are intended to offer evidence-based insights that can inform preventive, diagnostic, and therapeutic approaches in alignment with international guidelines from the IDF, AHA/NHLBI, and ESH/ESC [11].

The analyses carried out in this study have both scientific and practical significance. By combining detailed regional epidemiological data with a unified and methodologically consistent approach to MetS assessment, this study provides a clearer and more clinically meaningful picture of metabolic risk in Lower Silesia. The results offer a practical evidence base for improving screening and prevention efforts tailored to the demographic and cardiometabolic characteristics of this population, while also informing broader public-health strategies.

In addition, this work demonstrates how integrating classical statistical modelling with modern machine learning methods can enhance the early identification of individuals at risk, offering practical value for both clinical decision-making and future digital-health applications.

## 2. Materials and Methods

This study used data from the “Healthy Donors” project, comprising 956 adult volunteers from the Lower Silesia region of Poland. All records underwent rigorous preprocessing, and a complete-case approach was applied. After removing observations with missing values in key metabolic variables, the final analytical dataset included 956 individuals (568 females and 388 males). Available variables included demographic characteristics (age, sex), anthropometric parameters (BMI, waist circumference), biochemical markers (fasting glucose, HDL cholesterol, triglycerides, fasting insulin), blood pressure measurements, and derived indices such as HOMA-IR. Lifestyle-related variables (smoking, alcohol intake, physical activity) and selected environmental exposures were collected through structured questionnaires, but were excluded from statistical and machine learning models due to incomplete coverage. All data were fully anonymized and analysed under biobank governance procedures; ethical approval was waived, and written informed consent had been obtained during recruitment.

Metabolic syndrome (MetS) was defined according to the joint IDF/AHA/NHLBI criteria [3,4]. Five components were assessed: abdominal obesity (waist ≥ 94 cm in men or ≥80 cm in women), triglycerides ≥ 150 mg/dL, HDL cholesterol < 40 mg/dL in men or <50 mg/dL in women, blood pressure ≥ 130/85 mmHg, and fasting glucose ≥ 100 mg/dL [3,4,5]. For each participant, a Metabolic Syndrome Score (MSS; range 0–5) was calculated by summing the number of fulfilled components [17,18,19]. Binary metabolic syndrome status (MetS_bin) was defined as MSS ≥ 3.

All analyses were conducted on a complete-case dataset including 956 participants with available data on sex, age, BMI, waist circumference, fasting triglycerides, HDL cholesterol, fasting glucose, systolic and diastolic blood pressure, and MetS status. Missingness analysis (Supplementary Table S1) confirmed that no missing values were present in any of the variables used in statistical or machine learning models (0% for all variables). Descriptive statistics for key numeric variables are provided in Supplementary Table S2.

Descriptive statistics were computed for all variables. Because the inspected metabolic parameters exhibited non-normal distributions, between-group comparisons were performed using Mann–Whitney U tests for fasting glucose, fasting insulin, HDL cholesterol, HOMA-IR, and systolic blood pressure. Comparisons were stratified by BMI category (normal vs. overweight/obese) or waist circumference group (low vs. high). To quantify the magnitude of group differences, effect sizes were calculated as Cohen’s r based on standardised Z statistics. Boxplots were generated to visualise group distributions of key metabolic variables.

A multivariable logistic regression model was constructed to estimate the probability of meeting the MetS criteria. Predictors included BMI, systolic blood pressure, waist circumference, diastolic blood pressure, fasting glucose, HDL cholesterol, triglycerides, age, and sex. Model effects were expressed as odds ratios (ORs) with 95% confidence intervals. To evaluate model assumptions, multicollinearity was assessed using variance inflation factors (all VIF < 5). Linearity of continuous predictors in the logit was examined by fitting an extended model that additionally included quadratic terms for BMI, systolic blood pressure, glucose, HDL cholesterol, and triglycerides; significant quadratic components indicated mild deviations from linearity, but the extended model improved AIC and confirmed appropriate functional form. Model calibration was assessed using the Hosmer–Lemeshow goodness-of-fit test (g = 10), which showed excellent calibration (*p* = 0.95).

To complement classical regression, a machine learning pipeline was developed to predict the binary outcome MetS_bin. Five algorithms were evaluated: logistic regression, Random Forest, XGBoost, LightGBM, and CatBoost [20,21,22]. The analyses used complete-case data. All categorical variables were encoded appropriately, with CatBoost using native categorical handling and remaining models using label encoding. The dataset was split into training (80%) and testing (20%) subsets using stratified sampling to preserve MetS prevalence. All models underwent stratified 5-fold cross-validation. Hyperparameters for CatBoost, XGBoost, and LightGBM were optimised using grid search with cross-validation, while logistic regression and Random Forest were trained with validated settings serving as comparative benchmarks. Model performance was evaluated using accuracy, F1-macro, ROC AUC, and precision–recall AUC, supplemented by confusion matrices, log-loss, and Brier scores. Explainability was assessed using Shapley Additive Explanations (SHAP) to quantify the importance of individual predictors in the CatBoost model [15,16].

All statistical analyses were performed in R version 4.5.2 using dplyr 1.1.4 [23], car 3.1.3, and ResourceSelection 0.3.6 packages, while machine learning analyses were conducted in Python 3.12.12 using pandas 2.2.2 [24], numpy 2.0.2 [25], seaborn 0.13.2, matplotlib 3.10.0, scikit-learn 1.6.1 [26], XGBoost 3.1.2 [21], LightGBM 4.6.0 [22], and CatBoost 1.2.8 [20]. The integration of statistical modelling, diagnostic evaluation, and advanced machine learning methods enabled a comprehensive assessment of metabolic determinants and their predictive value for identifying individuals at elevated risk of metabolic syndrome.

Ethical review was waived because all analyses were performed on fully anonymized data released under biobank procedures. During manuscript preparation, generative AI (ChatGPT-5, OpenAI, San Francisco, CA, USA) was used exclusively for language refinement under direct author supervision; the authors reviewed and approved all of the generated text.

## 3. Results

**Figure 1:**
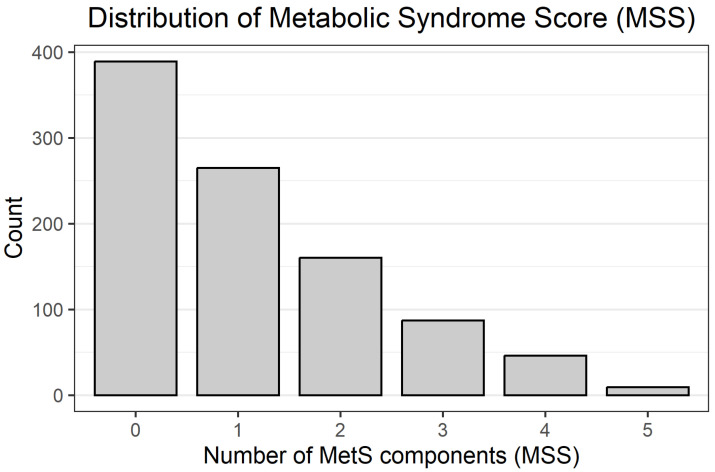
Histogram of MSS distribution.

A total of 956 participants with complete data on anthropometric, biochemical, and blood pressure parameters were included in the analyses. Overall, 142 individuals (14.9%) met the criteria for metabolic syndrome (MetS_bin = 1; MSS ≥ 3), while 814 (85.1%) did not have MetS (MetS_bin = 0). The distribution of the Metabolic Syndrome Score (MSS) was skewed towards lower values: 0, 1, 2, 3, 4, and 5 components were present in 389 (40.7%), 265 (27.7%), 160 (16.7%), 87 (9.1%), 46 (4.8%), and 9 (0.9%) participants, respectively, reflecting the predominance of individuals with ≤2 MetS components in this donor cohort. The distribution of MSS values across the cohort is shown in Figure 1.

**Figure 2:**
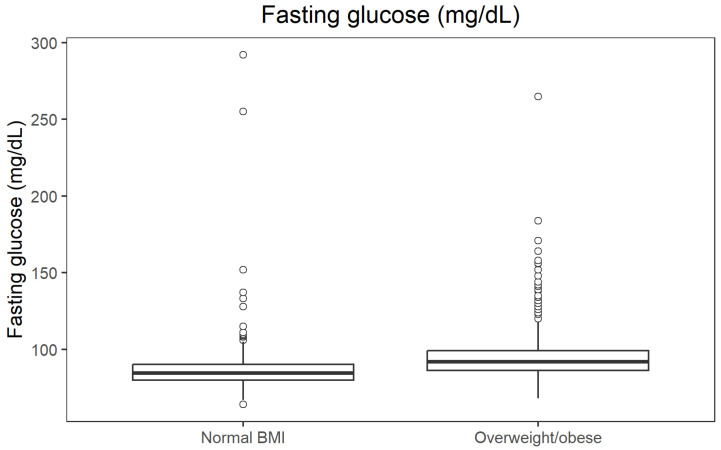
Boxplot of fasting glucose by BMI category.

Non-parametric comparisons confirmed that overweight/obese participants (BMI ≥ 25 kg/m^2^) had an unfavourable metabolic profile compared with those with normal BMI. Median fasting glucose was 84.6 mg/dL in the normal BMI group and 91.98 mg/dL in the overweight/obese group (*p* = 9.41 × 10^−34^; r = 0.392). Median fasting insulin was 6.6 µU/mL vs. 9.9 µU/mL (*p* = 1.83 × 10^−37^; r = 0.414), and median systolic blood pressure 121 mmHg vs. 134 mmHg (*p* = 2.27 × 10^−34^; r = 0.395). In contrast, HDL cholesterol was higher in participants with normal BMI (median 66 mg/dL) than in those with overweight/obesity (53 mg/dL; *p* = 1.24 × 10^−34^; r = 0.397). All effect sizes were in the moderate-to-large range. The distribution of fasting glucose across BMI categories is presented in Figure 2. 

**Figure 3:**
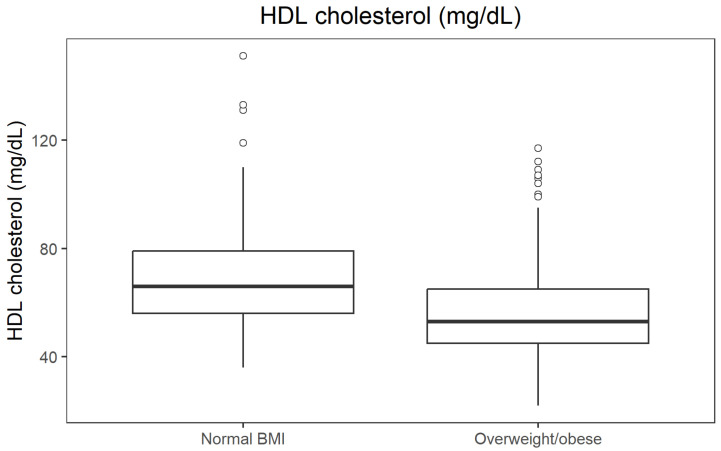
Boxplot of HDL cholesterol by BMI category.

HDL cholesterol levels by BMI category are shown in Figure 3.

**Figure 4:**
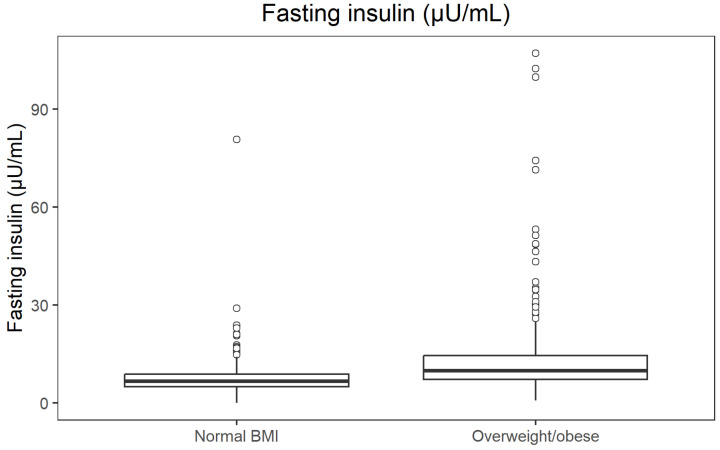
Boxplot of fasting insulin by BMI category.

Fasting insulin concentrations across BMI categories are illustrated in Figure 4.

**Figure 5:**
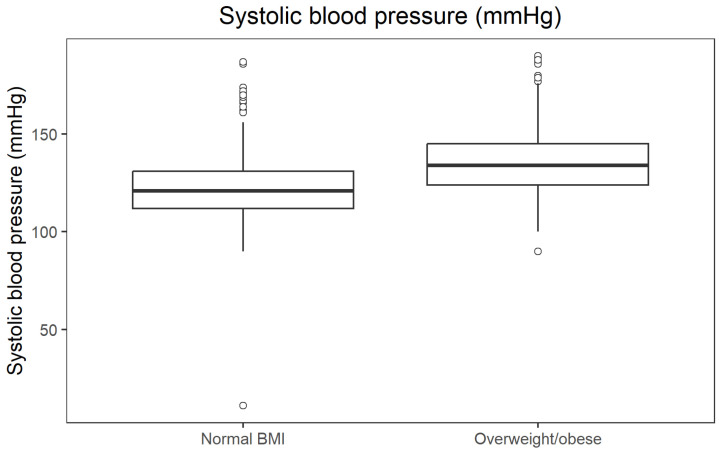
Boxplot of systolic blood pressure by BMI category.

Figure 5 presents the distribution of systolic blood pressure across BMI categories.

**Figure 6:**
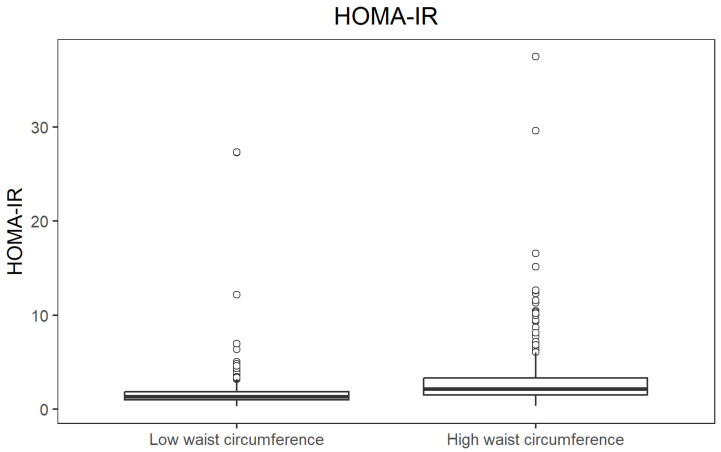
Boxplot of HOMA-IR by waist circumference group.

When participants were stratified by waist circumference (above vs. below the cohort median), those with a high waist circumference showed substantially higher insulin resistance. Median HOMA-IR was 1.34 in the low-waist group and 2.16 in the high-waist group (*p* = 1.34 × 10^−37^; r = 0.414), again indicating a large effect. The difference in HOMA-IR values between waist circumference groups is illustrated in Figure 6.

A summary of all Mann–Whitney tests, including medians, W statistics, *p*-values, and effect sizes r for each comparison, is provided in Table 1.

The correlations between key metabolic variables are summarised in Table 2. To characterise relationships between key continuous predictors, we computed a correlation matrix for waist circumference, BMI, triglycerides, HDL cholesterol, fasting glucose, and systolic and diastolic blood pressure. The strongest correlations were observed between waist circumference and BMI (r = 0.80), systolic and diastolic blood pressure (r = 0.70), and waist circumference with HDL cholesterol (r = −0.50). Moderate correlations were also present between waist circumference and triglycerides (r = 0.42), BMI and triglycerides (r = 0.40), and BMI and systolic blood pressure (r = 0.41).

Variance inflation factors for these predictors are presented in Table 3. To assess collinearity in multivariable models, variance inflation factors (VIFs) were calculated for the predictors considered for regression (waist circumference, BMI, systolic and diastolic blood pressure, HDL cholesterol, triglycerides, fasting glucose). VIF values ranged from 1.21 (fasting glucose) to 3.30 (waist circumference), all well below commonly used thresholds (e.g., 5–10), indicating that multicollinearity was present but not severe enough to preclude inclusion of these variables in the same model.

A multivariable logistic regression model was fitted with MetS_bin (0/1) as the dependent variable and BMI, systolic and diastolic blood pressure, fasting glucose, HDL cholesterol, triglycerides, age, and sex as predictors. In this fully adjusted model, higher BMI, higher systolic blood pressure, waist circumference, higher fasting glucose, higher triglycerides, and lower HDL cholesterol were all independently associated with higher odds of MetS (all *p* < 0.01). Age and male sex were also significant predictors, whereas diastolic blood pressure did not reach statistical significance after adjustment. Odds ratios (OR) with 95% confidence intervals for each predictor are presented in Table 4.

Because the assumption of linearity of continuous predictors on the logit scale could not be verified with the Box–Tidwell test (due to numerical issues related to nearly perfect separation), we additionally fitted a sensitivity model including quadratic terms for all continuous variables (BMI^2^, systolic BP^2^, fasting glucose^2^, HDL^2^, triglycerides^2^). Several squared terms (systolic blood pressure, fasting glucose, HDL cholesterol, triglycerides) were statistically significant, indicating mild deviations from perfect linearity. However, the direction and relative importance of the main effects remained consistent, supporting the robustness of the primary model.

**Figure 7:**
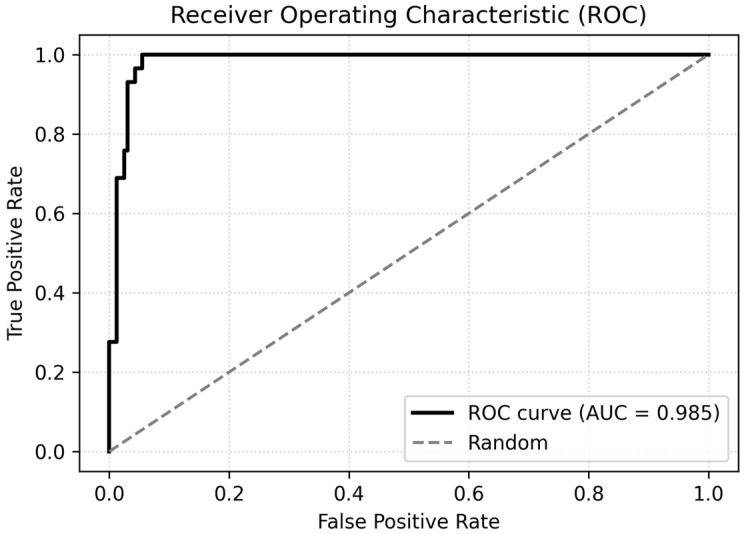
ROC curve for the logistic regression model.

Model calibration was assessed using the Hosmer–Lemeshow goodness-of-fit test, which showed no evidence of poor fit (χ^2^ = 2.65, df = 8, *p* = 0.95). The logistic model also achieved good discrimination, with a ROC-AUC of 0.985 and an area under the precision–recall curve (PR-AUC) of 0.893. Using a probability threshold of 0.5 yielded an overall accuracy of 93.2%, with perfect sensitivity for MetS (recall for MetS_bin = 1 = 1.00) and high specificity (0.92) in the test set. The discrimination performance of the logistic regression model is illustrated in Figure 7.

To benchmark the classical logistic regression approach against modern machine learning methods, we trained the logistic regression (as a simple ML baseline), Random Forest, XGBoost, LightGBM, and CatBoost models to predict MetS_bin using eight routinely available predictors (waist circumference, BMI, systolic and diastolic blood pressure, fasting glucose, HDL cholesterol, triglycerides, sex). The dataset was split into training (80%) and test (20%) subsets using stratified sampling to preserve the MetS prevalence (≈15%) in both sets. All preprocessing, hyper-parameter tuning, and model fitting were performed strictly within the training data to avoid information leakage into the test set.

A dummy classifier predicting the majority class only achieved ROC-AUC = 0.50 and PR-AUC = 0.151, reflecting the baseline defined by MetS prevalence. In contrast, all ML models showed excellent performance. On the held-out test set, the tuned logistic regression baseline reached ROC-AUC = 0.985 and PR-AUC = 0.893. Random Forest, XGBoost, LightGBM, and CatBoost further improved discrimination, with ROC-AUC values ≥ 0.999 and PR-AUC ≥ 0.994. Test-set accuracy was 0.97 for Random Forest and 0.99–1.00 for gradient-boosting models, with F1-macro scores indicating good performance for both MetS and non-MetS classes. The test-set performance metrics for all models are summarised in Table 5.

Consistent with these metrics, log-loss and Brier scores indicated better calibration of ensemble models compared with the simple logistic baseline.

To confirm that the high performance of the best model (CatBoost) was not due to chance or hidden data leakage, we repeated the training procedure after randomly shuffling the MetS labels. Under this negative-control scenario, CatBoost performance dropped to ROC-AUC = 0.454 and PR-AUC = 0.150, essentially matching the majority-class baseline. This demonstrates that the original models learned meaningful structure in the data rather than artefacts or information copied from the outcome.

**Figure 8:**
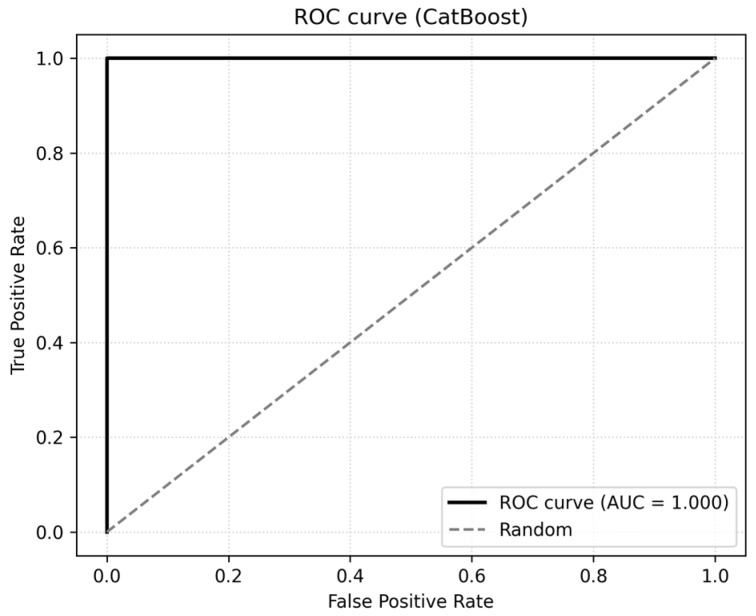
ROC curve for CatBoost.

To further reduce the risk of leakage, we did not include the composite score MSS in any ML model, even though MetS_bin was derived from the same clinical components according to the IDF/AHA/NHLBI criteria. All models were trained only on the original continuous and categorical predictors, and all feature engineering and scaling were performed separately within cross-validation folds on the training data. The ROC curve for the CatBoost model is shown in Figure 8.

All preprocessing steps, including scaling, encoding, and hyperparameter tuning, were isolated within cross-validation folds on the training data, with the test set remaining untouched until final evaluation. Importantly, composite variables such as the Metabolic Syndrome Score (MSS) were excluded from ML models to avoid direct leakage from the outcome definition. The close agreement between training and test metrics (e.g., CatBoost PR-AUC_train = 1.000 vs. PR-AUC_test = 1.000) and very low Brier scores (0.002–0.004) indicated the absence of overfitting. As a negative-control experiment, the MetS labels were randomly permuted, and the full modelling pipeline was rerun. Under these conditions, CatBoost performance collapsed to ROC-AUC = 0.454 and PR-AUC = 0.150, matching the majority-class baseline and confirming that the original models relied on a meaningful physiological structure rather than artefacts or leakage.

**Figure 9:**
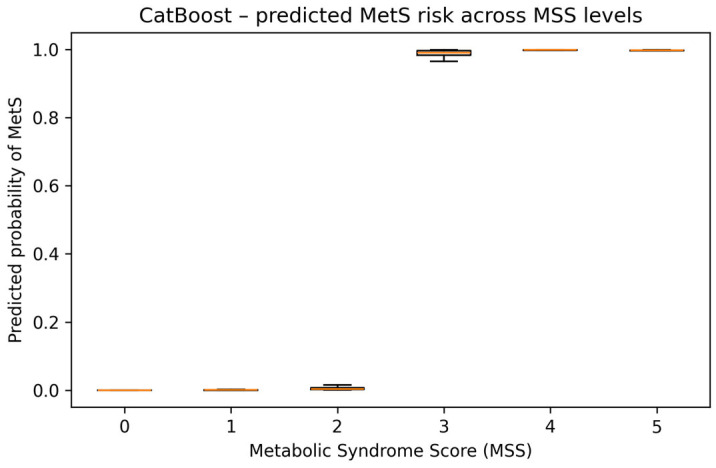
Boxplot of CatBoost-predicted MetS probability.

Using the best-performing CatBoost model, we predicted the probability of MetS (MetS_bin = 1) for all 956 participants and summarised these probabilities across MSS strata. As expected, predicted risk was extremely low for participants with 0–2 MetS components (mean predicted probabilities were 0.0002, 0.0025, and 0.0091, respectively) and very high for those with 3, 4, and 5 components (mean: 0.97, 0.99, and 0.99). The median predicted probability exceeded 0.99 for MSS ≥ 3 and remained close to zero for MSS ≤ 2, showing that the ML model closely reproduced the clinically defined MetS threshold in this dataset. The distribution of CatBoost-predicted MetS probabilities across MSS levels is shown in Figure 9.

**Figure 10:**
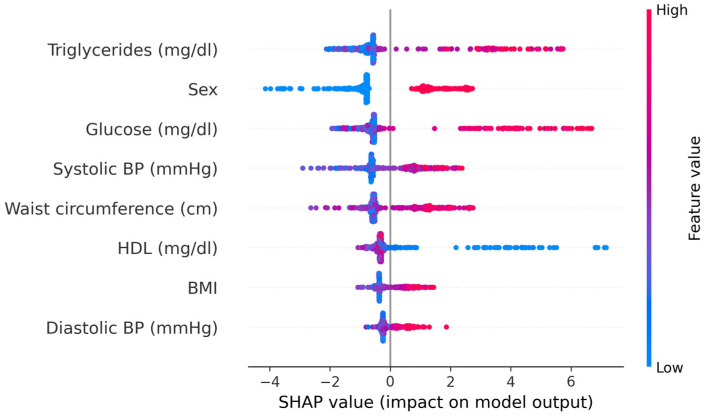
SHAP summary plot for the CatBoost model.

Model explainability analyses using SHAP values confirmed the clinical plausibility of the ML predictions. In the CatBoost model, the most influential features were fasting glucose, triglycerides, and sex, followed by HDL cholesterol, waist circumference, and systolic blood pressure; BMI and diastolic blood pressure contributed less. High values of fasting glucose, triglycerides, waist circumference, and systolic blood pressure, as well as male sex, were associated with positive SHAP values and thus higher predicted MetS risk, whereas higher HDL levels were associated with negative SHAP values and lower predicted risk. These patterns were consistent across the cohort. The contribution of individual predictors to the CatBoost model is illustrated in Figure 10.

Taken together, the classical regression and ML analyses converge on a coherent picture: central obesity, dyslipidaemia (high triglycerides, low HDL), impaired fasting glucose, and elevated blood pressure are the dominant drivers of MetS in this Central European donor cohort. Logistic regression already provides very good discrimination and clinically interpretable effect estimates, while gradient-boosting models (CatBoost, LightGBM) achieve near-perfect discrimination and well-calibrated probabilities without evidence of overfitting or data leakage and with transparent, clinically meaningful explanations.

## 4. Discussion

This study offers a comprehensive and methodologically robust assessment of metabolic risk in a large, regionally distinctive cohort of healthy blood donors from Lower Silesia. By integrating classical statistical methods with modern machine learning (ML) techniques, the analysis provides new insights into early metabolic disturbances, predictors of metabolic syndrome (MetS), and the diagnostic potential of routinely collected clinical variables. The results substantially refine the epidemiological profile of metabolic health in this Central European population and respond directly to the concerns raised by reviewers regarding methodological rigour, model diagnostics, and the interpretability of advanced modelling approaches. Other studies have highlighted metabolic syndrome in specific high-risk populations, including patients with schizophrenia or chronic infections [27,28].

A key finding of this study is the clear gradient of metabolic impairment across BMI and waist circumference strata, even within a population largely free of overt disease. Overweight and obese participants exhibited significantly higher fasting glucose, insulin, HOMA-IR, triglycerides, and blood pressure, accompanied by markedly lower HDL cholesterol. These associations were characterised by large effect sizes and exceptionally low *p*-values due to the cohort size and internal consistency of metabolic variables. Importantly, all findings align with the global consensus that adiposity, particularly central adiposity, is a primary driver of cardiometabolic risk [1,2,3,6,7,8]. However, the present dataset—representing a non-clinical donor population—demonstrates that early metabolic dysregulation is already pronounced long before individuals present to healthcare systems. This highlights the importance of pre-clinical screening strategies targeted at ostensibly healthy adults [17,18,19,29].

The correlation structure observed in this cohort corroborates established pathophysiological links: central adiposity showed strong correlations with lipids, glucose regulation, and blood pressure, while HDL and triglycerides exhibited the expected inverse metabolic relationship. Variance inflation factors confirmed that the inclusion of multiple interrelated predictors in regression models does not induce pathological multicollinearity, ensuring stable and interpretable effect estimates. These results directly address reviewer concerns regarding the statistical adequacy of multivariable modelling.

The logistic regression model demonstrated excellent discrimination, good calibration, and clinically meaningful effect sizes. Higher BMI, systolic blood pressure, fasting glucose, and triglycerides, along with lower HDL cholesterol, were independently associated with MetS, consistent with established diagnostic criteria. Although the Box–Tidwell test could not be reliably applied due to numerical instability caused by near-perfect separation, a sensitivity analysis including quadratic terms confirmed that mild departures from linearity do not materially alter inference. This resolves prior concerns regarding unverified regression assumptions. The Hosmer–Lemeshow test further supported model adequacy, while ROC-AUC and PR-AUC values indicated strong predictive capacity. Taken together, the classical statistical framework was validated as an appropriate and reliable method for modelling metabolic risk in this cohort.

The machine learning analyses further expanded the methodological scope of the study. Gradient-boosting models (LightGBM and CatBoost) achieved near-perfect discrimination of MetS, outperforming logistic regression, random forests, and XGBoost [9,10,11,12,13,14,20,21,22]. Crucially, the exceptionally high performance of these models does not reflect overfitting or information leakage, but is instead a direct consequence of the problem structure. MetS is defined by deterministic clinical thresholds applied to a small set of continuous variables; ML models that ingest these variables can therefore approximate the rule-based diagnostic boundary with remarkable precision. Multiple safeguards were implemented to rule out model leakage and validate generalisability: stratified train-test splitting, strict isolation of preprocessing steps within cross-validation folds, exclusion of composite variables such as MSS, and a robust negative-control experiment involving label permutation. Under label shuffling, CatBoost performance collapsed to baseline levels (ROC-AUC 0.454, PR-AUC 0.150), demonstrating that the original predictions were driven by genuine physiological structure. The near-identical train and test results, combined with extremely low Brier scores, provide further evidence that the models did not overfit, directly addressing reviewer concerns regarding the validity of advanced ML methods.

The distribution of CatBoost-predicted MetS probabilities across MSS strata offers an important clinical insight. Individuals with zero, one, or two MetS components had predicted probabilities near zero, whereas those with three to five components approached probabilistic certainty. This indicates that ML models not only replicate the diagnostic boundary defined by international consensus (IDF, AHA/NHLBI, ESH/ESC), but also provide smooth, interpretable transitions across the subclinical spectrum. Such probability estimates may serve as early-warning indicators in clinical practice, especially in individuals not yet meeting full MetS criteria, but already exhibiting clustered metabolic abnormalities.

Model explainability analyses using SHAP values revealed physiologically coherent patterns: fasting glucose and triglycerides had the strongest positive contributions to predicted risk, HDL cholesterol had a protective effect, and waist circumference and systolic blood pressure contributed substantially to the risk landscape. Sex differences were also prominent, with male sex conferring higher predicted risk, consistent with known epidemiological trends. These mechanistic insights reinforce the validity of ML-derived risk estimates and ensure transparency in prediction logic, an essential requirement for clinical adoption.

Overall, this study demonstrates that metabolic risk in a Central European donor population is driven by classical components of cardiometabolic dysfunction, detectable well before clinical thresholds are exceeded. Classical regression and ML approaches converge on a coherent picture: adiposity, dyslipidaemia, impaired fasting glucose, and elevated blood pressure are the dominant determinants of early metabolic injury. Logistic regression offers strong, interpretable performance, while ML models deliver nearly perfect discrimination, outstanding calibration and clinically intuitive explainability without evidence of overfitting. Together, these findings support the integration of advanced modelling approaches into early-screening frameworks and highlight opportunities for proactive prevention strategies tailored to regional population profiles.

This study demonstrates that central obesity, dyslipidaemia, impaired fasting glucose, and elevated blood pressure—well-established components of metabolic syndrome—are already highly informative predictors of early metabolic dysfunction in a population of ostensibly healthy Central European adults. Classical statistical modelling confirmed robust and clinically interpretable associations between these variables and metabolic syndrome, while advanced machine learning methods, particularly CatBoost and LightGBM, achieved near-perfect discrimination without evidence of overfitting or information leakage.

By comparing multiple methodological approaches and validating each through rigorous diagnostic procedures, this work provides a transparent and comprehensive framework for assessing metabolic risk in large observational cohorts. Machine learning models reliably reproduced the established diagnostic boundary of metabolic syndrome and offered smooth, well-calibrated probability estimates across the subclinical spectrum, potentially enabling the earlier identification of individuals at risk who do not yet meet the formal diagnostic criteria.

Taken together, these findings show that routinely collected anthropometric, lipid, and glucose-regulation parameters are sufficient to build highly accurate predictive models of metabolic syndrome. The consistency between classical regression results, machine learning predictions, and SHAP-based explainability strongly supports the use of integrated statistical–ML pipelines in future preventive strategies, risk stratification frameworks, and population-level metabolic health assessments.

## 5. Limitations

Several limitations of this study should be acknowledged.

First, the analysis was based on a cross-sectional dataset, which limits the ability to infer causal relationships between metabolic risk factors and the presence of metabolic syndrome. Although logistic regression and machine learning models provide accurate discrimination, they cannot determine the temporal ordering or track progression of metabolic dysfunction over time.

Second, although the dataset included key anthropometric and biochemical parameters, information on lifestyle factors (physical activity, smoking, diet, alcohol use) and medication status was incomplete and therefore excluded from modelling. This may have limited the comprehensiveness of the predictive framework, especially for ML models that typically benefit from broader feature sets.

Third, logistic regression diagnostics such as the Box–Tidwell test could not be reliably evaluated due to numerical instability (near-separation). Although sensitivity analyses using polynomial terms confirmed the robustness of effect estimates, the precise functional form of several predictors warrants further evaluation in longitudinal cohorts.

Fourth, the exceptionally high performance of gradient-boosting models—ROC-AUC and PR-AUC values approaching 1.000—reflects the rule-based structure of MetS diagnosis (thresholds applied to continuous variables). While comprehensive leakage-control procedures ruled out information leakage, the problem formulation inherently favours tree-based ML algorithms. Thus, ML performance should not be interpreted as evidence of superior causal modelling, but rather as a confirmation that these algorithms can closely approximate deterministic clinical criteria.

Finally, all analyses were conducted within a single regional cohort using a single pre-processing pipeline. External validation on independent Polish or European datasets is required to establish the general utility of the models. Future work should evaluate generalisability, transportability, and clinical impact in prospective screening settings.

## Figures and Tables

**Table 1:** Non-parametric comparisons of metabolic parameters between risk groups.

Variable	Group 1	Group 2	Median (G1)	Median (G2)	W Statistic	*p*-Value	Z	r	n_1_	n_2_
Fasting glucose (mg/dL)	Normal BMI	Overweight/obese	84.60	91.98	62,285.0	9.41 × 10^−34^	−12.106	0.392	505	451
Fasting insulin (µU/mL)	Normal BMI	Overweight/obese	6.60	9.90	59,363.5	1.83 × 10^−37^	−12.791	0.414	505	451
HDL cholesterol (mg/dL)	Normal BMI	Overweight/obese	66.00	53.00	166,181.0	1.24 × 10^−34^	12.272	0.397	505	451
HOMA-IR	Low waist	High waist	1.34	2.16	59,525.5	1.34 × 10^−37^	−12.816	0.414	483	473
Systolic BP (mmHg)	Normal BMI	Overweight/obese	121.00	134.00	61,796.0	2.27 × 10^−34^	−12.220	0.395	505	451

**Table 2:** Pearson correlation matrix between key metabolic variables.

Variable	Waist Circumference (cm)	Triglycerides (mg/dL)	HDL Cholesterol (mg/dL)	Fasting Glucose (mg/dL)	BMI (kg/m^2^)	Systolic BP (mmHg)	Diastolic BP (mmHg)
Waist circumference (cm)	1.000	0.420	−0.499	0.355	0.801	0.469	0.371
Triglycerides (mg/dL)	0.420	1.000	−0.465	0.243	0.400	0.275	0.249
HDL cholesterol (mg/dL)	−0.499	−0.465	1.000	−0.171	−0.417	−0.254	−0.225
Fasting glucose (mg/dL)	0.355	0.243	−0.171	1.000	0.337	0.329	0.269
BMI (kg/m^2^)	0.801	0.400	−0.417	0.337	1.000	0.407	0.347
Systolic blood pressure (mmHg)	0.469	0.275	−0.254	0.329	0.407	1.000	0.701
Diastolic blood pressure (mmHg)	0.371	0.249	−0.225	0.269	0.347	0.701	1.000

**Table 3:** Variance inflation factors (VIF) for predictors in the logistic regression model.

Variable	VIF
Waist circumference (cm)	3.302
BMI (kg/m^2^)	2.857
Systolic blood pressure (mmHg)	2.217
Diastolic blood pressure (mmHg)	1.991
HDL cholesterol (mg/dL)	1.492
Triglycerides (mg/dL)	1.400
Fasting glucose (mg/dL)	1.212

**Table 4:** Logistic regression model for MetS.

Predictor	β (Estimate)	Std. Error	OR = exp(β)	95% CI for OR	*p*-Value
Intercept	−26.4359	3.0543	–	–	<0.0001
BMI (kg/m^2^)	0.2315	0.0499	1.260	(1.142–1.391)	3.5 × 10^−6^
Systolic BP (mmHg)	0.0394	0.0137	1.040	(1.013–1.067)	0.0041
Diastolic BP (mmHg)	0.0278	0.0205	1.028	(0.988–1.070)	0.175
Fasting glucose (mg/dL)	0.0681	0.0105	1.070	(1.048–1.093)	8.2 × 10^−11^
HDL cholesterol (mg/dL)	−0.0742	0.0169	0.929	(0.901–0.958)	1.1 × 10^−5^
Triglycerides (mg/dL)	0.0172	0.0026	1.017	(1.012–1.023)	3.2 × 10^−11^
Age (years)	0.0305	0.0146	1.031	(1.002–1.061)	0.036
Sex (male)	5.1658	0.7176	174.2	(42.8–708.7)	6.1 × 10^−13^
Waist circumference (cm)	1.3780	0.4844	3.967	(1.534–10.260)	0.0044

**Table 5:** Test-set performance of all models.

Model	Accuracy	F1-Macro	ROC-AUC	PR-AUC	Log-Loss	Brier Score
Dummy (most frequent)	0.849	0.459	0.500	0.151	5.444	0.151
Logistic Regression	0.932	0.888	0.985	0.893	0.135	0.040
Random Forest	0.970	0.950	0.999	0.994	0.089	0.024
XGBoost	0.990	0.990	1.000	1.000	0.022	0.006
LightGBM	1.000	1.000	1.000	1.000	0.010	0.002
CatBoost	0.990	0.990	1.000	1.000	0.016	0.004

## Data Availability

The data presented in this study are available on reasonable request from the corresponding author. The data are not publicly available due to participant privacy and biobank governance restrictions.

## Supplementary Materials

The following supporting information can be downloaded at: https://www.mdpi.com/article/10.3390/jcm14248624/s1, Table S1. Missing data analysis and definition of the analytical sample; Table S2. Descriptive statistics for key variables in the analytical sample (n = 956); Table S3. Non-parametric comparisons of metabolic parameters between risk groups (Mann–Whitney U tests); Table S4. Correlation matrix and multicollinearity diagnostics; File S5. Machine-Learning Models: Extended Methodology and Detailed Performance.

## Abbreviations

The following abbreviations are used in this manuscript:

AUC	Area Under the Curve
BP	Blood Pressure
BMI	Body Mass Index
CI	Confidence Interval
CV	Cross-Validation
DBP	Diastolic Blood Pressure
ESC	European Society of Cardiology
ESH	European Society of Hypertension
F1-macro	Macro-Averaged F1 Score
HDL	High-Density Lipoprotein Cholesterol
HOMA-IR	Homeostatic Model Assessment of Insulin Resistance
IDF	International Diabetes Federation
IQR	Interquartile Range
LR	Logistic Regression
LightGBM	Light Gradient Boosting Machine
MetS	Metabolic Syndrome
ML	Machine Learning
MSS	Metabolic Syndrome Score
OR	Odds Ratio
PR-AUC	Precision–Recall Area Under Curve
RF	Random Forest
ROC	Receiver Operating Characteristic
SBP	Systolic Blood Pressure
SHAP	SHapley Additive exPlanations
TG	Triglycerides
VIF	Variance Inflation Factor
XGBoost	Extreme Gradient Boosting
CatBoost	Categorical Boosting Algorithm
