# Supplementary material for: Risk Analysis in the Lower Silesia Healthy Donors Cohort: Statistical Insights and Machine Learning Classification
Source: J Clin Med. 2025 Dec 5;14(24):8624. doi: 10.3390/jcm14248624 (PMC12733403; doi:10.3390/jcm14248624)
Supplement: Supplementary file 1 [file jcm-14-08624-s001.zip › jcm-4003481-supplementary.pdf]

Supplementary Table S1. Missing data analysis and definition of the analytical sample

S1A. Missingness in the full cohort (n = 1151)

Variable	n non-missing	n missing	Missing %
**BMI (kg/m²)**	1151	0	0.0%
**Waist circumference (cm)**	1128	23	2.0%
**Triglycerides (mg/dL)**	1151	0	0.0%
**HDL cholesterol (mg/dL)**	1151	0	0.0%
**Fasting glucose (mg/dL)**	1151	0	0.0%
**Fasting insulin (µU/mL)**	1151	0	0.0%
**HOMA-IR**	1150	1	0.1%
**Blood pressure value (mmHg, "SBP/DBP")**	968	183	15.9%

S1B. Missingness in the analytical sample (complete-case dataset; n = 956)

The analytical sample was obtained by restricting the full dataset (n = 1151) to observations with non-missing values in all key metabolic variables:

- BMI
- Waist circumference
- Triglycerides
- HDL cholesterol
- Fasting glucose
- Fasting insulin
- HOMA-IR
- Blood pressure value ("SBP/DBP")

After complete-case filtering, **956 participants** remained. For all variables used in statistical and machine-learning models, the proportion of missing values was **0% (n missing = 0 for all variables)**.

Variable	n non-missing	n missing	Missing %

**BMI (kg/m^2)**	956	0	0.0%
**Waist circumference (cm)**	956	0	0.0%
**Triglycerides (mg/dL)**	956	0	0.0%
**HDL cholesterol (mg/dL)**	956	0	0.0%
**Fasting glucose (mg/dL)**	956	0	0.0%
**Fasting insulin (μU/mL)**	956	0	0.0%
**HOMA-IR**	956	0	0.0%
**Systolic blood pressure (mmHg)**	956	0	0.0%
**Diastolic blood pressure (mmHg)**	956	0	0.0%
**MSS (0–5)**	956	0	0.0%
**MetS_bin (0/1)**	956	0	0.0%

Supplementary Table S2. Descriptive statistics for key variables in the analytical sample (n = 956)

Values are based on the analytical dataset (dane_model), which includes 956 complete cases.

**Variable**	**Median**	**IQR (Q3–Q1)**	**Min**	**Max**
**Age (years)**	40.0	25.0	17.0	80.0
**BMI (kg/m^2)**	24.77	6.26	0.00*	44.98
**Waist circumference (cm)**	86.0	22.0	32	134
**Triglycerides (mg/dL)**	88.5	68.0	31	737
**HDL cholesterol (mg/dL)**	60.0	23.0	22	151
**Fasting glucose (mg/dL)**	88.02	13.14	64.08	291.96

**Systolic blood pressure (mmHg)**	127	21.0	11†	190
**Diastolic blood pressure (mmHg)**	79	15.0	47	112
**MSS (number of MetS components)**	1	2	0	5

* One extreme BMI value of 0.00 kg/m^2 is present in the original dataset; this value was retained in the analytical sample.
† One extreme systolic value (11 mmHg) appears in the source data; no additional truncation was applied at the preprocessing stage.

The distribution of the Metabolic Syndrome Score (MSS, 0–5) in the analytical sample was:

- MSS = 0: 389 (40.7%)
- MSS = 1: 265 (27.7%)
- MSS = 2: 160 (16.7%)
- MSS = 3: 87 (9.1%)
- MSS = 4: 46 (4.8%)
- MSS = 5: 9 (0.9%)

Binary MetS status (MetS_bin) was defined as MSS ≥ 3:

- MetS_bin = 0: 814 participants (85.1%)
- MetS_bin = 1: 142 participants (14.9%)

Supplementary Table S3. Non-parametric comparisons of metabolic parameters between risk groups (Mann–Whitney U tests)

This table summarises the Wilcoxon–Mann–Whitney tests reported in the main text. Effect size r was calculated as $|Z| / \sqrt{(n_1 + n_2)}$.

Variable	Grouping variable	Group 1 (reference)	Group 2	Median G1	Median G2	W statistic	p-value	Z	r effect	n_1	n_2
**Fasting glucose (mg/dL)**	BMI group	Normal BMI	Overweight/obese	84.60	91.98	62,285.0	9.41×10^{-34}	−12.106	0.392	505	451
**Fasting insulin (μU/mL)**	BMI group	Normal BMI	Overweight/obese	6.60	9.90	59,363.5	1.83×10^{-37}	−12.791	0.414	505	451

**HDL cholesterol (mg/dL)**	BMI group	Normal BMI	Overweight/obese	66.00	53.00	166,181.0	1.24×10^{-34}	12.272	0.397	505	451
**HOMA-IR**	Waist circumference	Low waist circumference	High waist circumference	1.34	2.16	59,525.5	1.34×10^{-37}	–12.816	0.414	483	473
**Systolic BP (mmHg)**	BMI group	Normal BMI	Overweight/obese	121.0	134.0	61,796.0	2.27×10^{-34}	–12.220	0.395	505	451

Supplementary Table S4. Correlation matrix and multicollinearity diagnostics

S4A. Pearson correlation matrix for key metabolic predictors (analytical sample, n = 956)

Variable	Waist circ.	Triglycerides	HDL chol.	Fasting glucose	BMI	Systolic BP	Diastolic BP
**Waist circumference (cm)**	1.000	0.420	–0.499	0.355	0.801	0.469	0.370
**Triglycerides (mg/dL)**	0.420	1.000	–0.464	0.243	0.400	0.275	0.248
**HDL cholesterol (mg/dL)**	–0.499	–0.464	1.000	–0.170	–0.417	–0.254	–0.225
**Fasting glucose (mg/dL)**	0.355	0.243	–0.170	1.000	0.337	0.329	0.268
**BMI (kg/m²)**	0.801	0.400	–0.417	0.337	1.000	0.407	0.346
**Systolic blood pressure (mmHg)**	0.469	0.275	–0.254	0.329	0.407	1.000	0.701
**Diastolic blood pressure (mmHg)**	0.370	0.248	–0.225	0.268	0.346	0.701	1.000

The strongest correlations were observed between waist circumference and BMI ($r \approx 0.80$), and between systolic and diastolic blood pressure ($r \approx 0.70$). As expected, HDL cholesterol was negatively correlated with triglycerides, waist circumference, BMI and blood pressure.

Supplementary File S5. Machine-Learning Models: Extended Methodology and Detailed Performance

This supplementary section provides full details on the machine-learning pipeline, hyperparameter tuning procedures, and complete performance metrics for all evaluated models. The information extends Table 5 in the main manuscript.

S5A. Dataset, Outcome Definition and Baseline Model

Dataset and outcome:

- Analytical dataset size: n = 956
- Outcome: MetS_bin (0/1), defined as Metabolic Syndrome presence according to IDF/AHA/NHLBI criteria (MSS ≥ 3)
- Predictors used consistently across all machine-learning models:
  • Waist circumference (cm)
  • BMI (kg/m²)
  • Systolic blood pressure (mmHg)
  • Diastolic blood pressure (mmHg)
  • Fasting glucose (mg/dL)
  • HDL cholesterol (mg/dL)
  • Triglycerides (mg/dL)
  • Sex

Train/test split:

- Train set: n = 764 (80%)
- Test set: n = 192 (20%)
- Stratified by MetS prevalence (train: 0.148; test: 0.151)

Baseline model – Dummy classifier (most frequent class):

- Accuracy: 0.849
- F1-macro: 0.459
- ROC-AUC: 0.500
- PR-AUC: 0.151
- Log-loss: 5.444
- Brier score: 0.151

This represents the minimum performance threshold for a model to be considered useful.

S5B. Logistic Regression (Machine-Learning Baseline)

Model:
Penalised logistic regression (scikit-learn). Standardization applied using StandardScaler within cross-validation folds.

Hyperparameter tuning (GridSearchCV, 5-fold CV):

- C: 0.01, 0.1, 1.0, 10.0
- Penalty: L2

- Solver: lbfgs, liblinear, saga

Best hyperparameters (ROC-AUC optimisation):

- C = 10.0

Best hyperparameters (PR-AUC optimisation):

- C = 0.1, penalty = L2, solver = lbfgs
- Best cross-validated PR-AUC ≈ 0.872

Test-set performance:

- ROC-AUC: 0.985
- PR-AUC: 0.893
- Accuracy: 0.932
- F1-macro: 0.888
- Log-loss: 0.135
- Brier score: 0.040

Confusion matrix (threshold 0.5):
True 0: 150 predicted as 0, 13 predicted as 1
True 1: 0 predicted as 0, 29 predicted as 1

S5C. Random Forest

Hyperparameter grid:

- n_estimators: 200, 400, 600
- max_depth: None, 4, 6
- min_samples_split: 2, 5

Best hyperparameters:

- n_estimators = 600
- max_depth = None
- min_samples_split = 2
- Best cross-validated PR-AUC ≈ 0.969

Test-set performance:

- ROC-AUC: 0.999
- PR-AUC: 0.994
- Accuracy: 0.970

- F1-macro: 0.950
- Log-loss: 0.089
- Brier score: 0.024

Confusion matrix:
True 0: 163 predicted as 0, 0 predicted as 1
True 1: 5 predicted as 0, 24 predicted as 1

S5D. CatBoost

Hyperparameter grid:

- n_estimators: 200, 300, 500, 800
- depth: 4, 6, 8
- learning_rate: 0.03, 0.05, 0.1
- l2_leaf_reg: 3, 5, 7

Best hyperparameters:

- n_estimators = 300
- depth = 6
- learning_rate = 0.05
- l2_leaf_reg = 3
- Best cross-validated PR-AUC ≈ 0.995

Test-set performance:

- ROC-AUC: 1.000
- PR-AUC: 1.000
- Accuracy: 0.990
- F1-macro: 0.990
- Log-loss: 0.016
- Brier score: 0.004

Confusion matrix:
True 0: 163 predicted as 0, 0 predicted as 1
True 1: 1 predicted as 0, 28 predicted as 1

S5E. XGBoost

Hyperparameter grid:

- n_estimators: 200, 400, 600

- max_depth: 3, 4, 5
- learning_rate: 0.01, 0.05, 0.1
- subsample: 0.6, 0.8, 1.0
- colsample_bytree: 0.8, 1.0

Best hyperparameters:

- n_estimators = 600
- max_depth = 3
- learning_rate = 0.05
- subsample = 0.8
- colsample_bytree = 1.0
- Best cross-validated PR-AUC ≈ 0.996

Test-set performance:

- ROC-AUC: 1.000
- PR-AUC: 1.000
- Accuracy: 0.990
- F1-macro: 0.990
- Log-loss: 0.022
- Brier score: 0.006

Confusion matrix:
True 0: 163 predicted as 0, 0 predicted as 1
True 1: 1 predicted as 0, 28 predicted as 1

S5F. LightGBM

Hyperparameter grid:

- n_estimators: 500, 1000
- max_depth: -1, 5, 7
- num_leaves: 31, 63
- learning_rate: 0.05, 0.1
- subsample: 0.8, 1.0
- colsample_bytree: 0.8, 1.0

Best hyperparameters:

- n_estimators = 1000
- max_depth = 5
- num_leaves = 31
- learning_rate = 0.1
- subsample = 1.0
- colsample_bytree = 0.8
- Best cross-validated PR-AUC ≈ 0.991

Test-set performance:

- ROC-AUC: 1.000
- PR-AUC: 1.000
- Accuracy: 1.000
- F1-macro: 1.000
- Log-loss: 0.010
- Brier score: 0.002

Confusion matrix:
True 0: 163 predicted as 0, 0 predicted as 1
True 1: 0 predicted as 0, 29 predicted as 1
